# The Impact of Antiretroviral Therapy on Liver Function Among Pregnant Women Living with HIV in Co-Existence with and Without Pre-Eclampsia

**DOI:** 10.3390/v17010028

**Published:** 2024-12-28

**Authors:** Kay-Lee E. Strauss, Wendy N. Phoswa, Kabelo Mokgalaboni

**Affiliations:** Department of Life and Consumer Sciences, College of Agriculture and Environmental Sciences, Florida Campus, Roodepoort 1709, South Africa; 68188242@mylife.unisa.ac.za (K.-L.E.S.); phoswwn@unisa.ac.za (W.N.P.)

**Keywords:** antiretroviral therapy, liver function, human immunodeficiency virus, pre-eclampsia, pregnancy

## Abstract

Pregnant women living with HIV (PWLWHIV) are at an increased risk of developing obstetrics complications such as pre-eclampsia (PE). Antiretroviral therapy (ART) remains the standard treatment for PWLWHIV and non-pregnant women. However, its use has been associated with adverse liver conditions, particularly hepatotoxicity, often marked by elevated liver enzymes (LEEs) as demonstrated by an increased aspartate transferase (AST), alanine transaminase (ALT), and alkaline phosphatase (ALP) in PWLWHIV on ART. Morever, there is limited evidence about the effect of ART on liver function among PWLWHIV and PE. Therefore, this review examines the pathogenesis of PE and the impact of ART on liver function in PWLWHIV with and without PE. With the evidence gathered in this review, it is still unclear whether liver dysfunctions in PWLWHIV in co-existence with orwithout PE result from HIV infection or ART administration or are exacerbated by the presence of PE. Among those without PE, there was an increase in liver enzymes, a decrease, and no effect in other studies in ART-treated PWLWHIV compared to the control group. Additionally, among those with PE, the impact of ART remains unclear due to contradicting results. The notable trend was that nevirapine was associated with a reduced risk of liver dysfunction among PWLWHIV without PE. Therefore, more studies are needed in this area, especially in HIV endemic regions, to understand the exact cause of liver dysfunction in this population. This knowledge is crucial for improving liver function and PE management among PWLWHIV.

## 1. Introduction

In 2020, the World Health Organisation (WHO) reported that about 800 women lost their lives daily due to avoidable factors associated with pregnancy and childbirth [1]. The primary factors responsible for about 75% of all maternal fatalities include excessive haemorrhage, postpartum infections, complicated delivery, botched abortion, pre-eclampsia (PE), and eclampsia [1]. One of the significant and potentially life-threatening pregnancy-related complications is PE, affecting approximately 2–10% of pregnancies worldwide [2]. Despite regional variations in PE rates, sub-Saharan Africa (SSA) reports an overall prevalence of 13%, above the global average [3]. In South Africa, the prevalence of PEis reportedly standing at 5.7% [4]. These rising rates warrant a need for enhanced maternal healthcare systems, especially in areas with higher prevalence rates, to reduce the complications associated with PE.

PE is a gestational condition characterised by proteinuria and hypertension, thrombocytopenia, and organ damage after 20 weeks of gestation [5]. While treatment options are available for pregnant women with hypertension, there is still no definitive cure for PE [6]. The early delivery of the faulty placenta and baby before the gestational period is frequently used as a method to remedy the issue [7]. This, however, can lead to severe complications for the foetus, including reduced birth weight, restricted foetal growth, and smaller for gestational age [8]. Moreover, inadequate blood flow and impaired placental function can result in intrauterine growth restriction, premature birth [9], and, in severe cases, stillbirth [10]. These factors contribute to chronic health complications for the child [11]. On the other hand, the maternal complications of PE can be severe, including prolonged hypertension, which subsequently leads to eclampsia; HELLP syndrome (haemolysis, elevated liver enzymes, and low platelet count); and organ damage [12]. Therefore, it is important that healthcare providers closely monitor and manage PE to prevent these severe complications for both the mother and the infant. Early detection and appropriate treatment of PE can prevent eclampsia and subsequent complications thereof.

The cause of PE is multifaceted [13]. Other researchers have suggested its pathogenesis may be associated with inadequate placentation due to an impaired immune system [14]. Moreover, genetic variations have also been reported as contributing factors that can impact different physiological pathways, including those involved in regulating blood pressure, endothelial function, and the inflammatory response [15]. The complex and multifaceted aetiology of PE makes it difficult to manage effectively. This becomes worse when the pregnant woman is also infected with HIV. A South African study conducted in 2013 revealed that 26.4% of women with PE were living with HIV when compared to 36.6% in the control group [16]. As most PWLWHIV rely on ART, it is worth noting that some of these ART regimens may interfere with uteroplacental blood flow, resulting in insufficient placental perfusion, a central feature of PE [17,18,19]. Tooke et al. 2016 showed that ART duration (4 weeks and above) is associated with the development of severe PE [20]. Notably, PE impairs liver function, resulting in elevated liver enzymes (LEE), such as aspartate aminotransferase (AST) and alanine aminotransferase (ALT). This elevation is modulated by endothelial dysfunction, which induces hepatic hypoxia and necrosis of hepatocytes [21,22]. Therefore, monitoring liver function frequently in PWLWHIV in co-existence with PE is crucial to improving the health of the foetus and mother during pregnancy. ART is associated with placental alterations, including increased placental oxidative stress and vascular resistance, which may predispose pregnant women to PE. This review examines the pathogenesis of PE and the effect of ART on liver function in PWLWHIV in co-existence with or without PE.

### 1.1. Inflammatory Response and the Pathogenesis of Pre-Eclampsia

The widespread inflammation and impaired endothelium function is a well-known characteristic of PE [23,24]. The inflammatory response is a crucial factor in the progression of the disease and has an impact on multiple organs, including the liver [25]. Immunological imbalances in mothers, especially related to innate immunity, play a role in initiating the immune response linked to the development of PE. Alterations in the mononuclear phagocyte system substantially impact the inflammatory response [26]. Due to the hypoxic nature of the placenta, pro-inflammatory cytokines, such as tumour necrosis factor-alpha (TNF-α), interleukin-6 (IL-6), and interleukin-1 beta (IL-1β), and anti-angiogenic factors, like soluble fms-like tyrosine kinase-1 (sFLT-1) and soluble endoglin, are released into the maternal bloodstream [27]. Oxidative stress, peripheral blood mononuclear cells, macrophages, and endothelial and vascular smooth muscle cells could cause sFLT-1 levels to rise in PE cases [28]. Research indicates that generalised endotheliosis in the systemic, renal, cerebral, and hepatic circulation may reduce the production of vasodilators such as nitric oxide, prostacyclin, and the hyperpolarisation factor [29]. This decrease can cause vasoconstrictors like endothelin-1 (ET-1) and thromboxane A2 to increase, resulting in vasoconstriction and hypertension [30]

Moreover, the imbalance between vasodilators and vasoconstrictors can decrease blood flow to the placenta, which can then cause the release of sFLT-1 into the mother’s bloodstream [31]. The excess sFLT-1 attaches to and hinders the action of vascular endothelial growth factor (VEGF) and placental growth factor (PlGF), intensifying the vasoconstriction and endothelial dysfunction observed in PE [31,32]. Overall, the intricate interaction of multiple elements results in the clinical symptoms of PE.

Due to its extensive blood supply and crucial role in the body’s metabolism and immune response, the liver is vulnerable to inflammation and impaired function of the blood vessels lining the liver in PE [24]. PE causes systemic endothelial dysfunction, which affects the hepatic vasculature [33]. Inflammation-inducing cytokines and substances that inhibit the growth of blood vessels damage the cells that line the hepatic sinusoids, resulting in reduced blood flow and a lack of oxygen in liver tissues [34]. Oxidative stress caused by the hypoxic conditions in PE leads to liver cell damage [35]. Hepatocyte injury and death can occur due to the presence of reactive oxygen species (ROS) resulting from hypoxia and inflammation [36]. This leads to the release of liver enzymes, including ALT and AST, into the bloodstream [37]. Pro-inflammatory cytokines such as TNF-α can directly trigger the death of liver cells through apoptosis or necrosis [38]. The cellular damage causes liver enzymes to flow into the maternal bloodstream, indicating hepatic injury [39].

PE triggers an inflammatory response that causes widespread malfunction of endothelial cells throughout the body, impairing multiple organs, including the liver [18,40]. Specific ART regimens, especially those incorporating protease inhibitors (PIs), have been linked to metabolic and vascular alterations, such as endothelial injury, insulin resistance, and dyslipidaemia, risk factors for PE [41]. The increased activity of liver enzymes in PE is caused by damage to the liver’s endothelial cells, oxidative stress, and a direct impact on liver cells caused by inflammation. Hence, understanding this relationship is essential for efficient control and management of PE and its associated complications.

### 1.2. Genetic Factors and the Pathogenesis of Pre-Eclampsia

Genetic predisposition is a significant factor in the onset of PE, and all the components associated with the development of PE have hereditary variables that may contribute to the pathological manifestations [42]. A study conducted by Bezerra et al. (2010) found that having a family history of hypertensive disorders increases the chance of developing eclampsia and HELLP syndrome [43]. Focusing on genetic aspects such as familial clustering, candidate genes, and genomic investigation is important when studying PE. Incorporating genetic factors into the study of PE is important to understand and monitor maternal and foetal health.

PE has familial patterns, indicating a genetic contribution to its aetiology [44]. A previous study found that maternal and paternal early-onset chronic hypertension and paternal early-onset myocardial infarction were independent risk factors for severe PE [45]. A positive family history of cardiovascular disorders before the age of 50 increased the risk of early-onset PE by 5.05-fold compared to the control group. The results suggest that familial early-onset cardiovascular disorders are a predisposing factor for severe PE [45]. Another study found that PE in daughters is associated with increased risks of cardiovascular disease (CVD) in parents [46]. The study showed that parents having one daughter with PE were 1.19 times more likely to develop CVD at age under 55 years. The study suggests that PE and CVD share common heritable mechanisms [46]. These findings emphasise the significance of considering familial history when evaluating the likelihood of getting severe PE. Additional investigation into the common heritable processes underlying PE and CVD may result in novel understandings and viable therapies for these disorders.

Several genes have been studied for their potential involvement in the development of PE. These genes are associated with pathways linked to angiogenesis, immunological response, oxidative stress, and blood pressure regulation [47]. For example, the vascular endothelial growth factor A (VEGFA) gene encodes VEGFA, which is involved in angiogenesis [48]. Therefore, dysfunction in the VEGFA gene may contribute to the development of PE. Additionally, the expression of placental sFLT-1, an inhibitor of VEGF and placental growth factor, is raised in cases of PE [31]. This results in elevated levels of sFLT-1 in the bloodstream, which decreases after delivery. On the other hand, the angiotensin-converting enzyme (ACE) gene encodes an enzyme that controls blood pressure and electrolyte balance by converting angiotensin I into angiotensin II, which narrows blood vessels and stimulates aldosterone production [49]. Genetic variations substantially affect ACE levels [50]. Thereby resulting in impaired production of angiotensin II, contributing to hypertension and related cardiovascular-related complications [51]. Conversely, TNF genes encode tumour necrosis factor-alpha, a crucial pro-inflammatory cytokine [52]. An increased level of TNF-α is associated with inflammation and oxidative stress, both of which contribute to the development of PE [25]. These genetic variations play a significant role in the development and progression of PE.

Genetic predispositions can amplify inflammatory reactions, impairing endothelium and organ damage, especially in the liver [53]. The variations in genes encoding pro-inflammatory cytokines can make individuals more susceptible to increased inflammatory reactions [54]. For instance, TNF-α and IL-6 can enhance inflammatory reactions and result in adverse pregnancy complications, including PE [55]. The changes in genes associated with oxidative stress can also impair the body’s capacity to regulate ROS, resulting in cellular damage and inflammation [56]. It has been shown that the glutathione S-transferase (GST) and superoxide dismutase (SOD) genes can result in an imbalance between enzymes that remove ROS, resulting in oxidative stress and inflammation [57]. On the other hand, the genes involved in angiogenesis can shift the balance between molecules that promote angiogenesis and those that inhibit angiogenesis [58]. This can affect the development of the placenta and, further, promote inflammation. The polymorphism of VEGFA and FLT1 changes the expression of the VEGF gene, resulting in hypoxia and inflammation in PE [32]. FLT-1 gene variants also raise the soluble FLT-1, which leads to endothelial dysfunction and inflammation in PE [59].

A multicentric meta-analysis of 20,064 cases and 703,117 control individuals revealed the presence of 18 independent loci associated with PE, eclampsia, and gestational hypertension [60]. These genetic locations emphasise the importance of natriuretic peptide signalling, angiogenesis, renal glomerular function, trophoblast development, and immune dysregulation. Another genome-wide association study (GWAS) that examined the relationship between PE and maternal hypertension in pregnancy identified at least 19 significant connections, 13 of which were previously unknown [15]. Genes related to blood pressure features were connected to seven new loci. The analysis discovered new risk locations. These findings offer a detailed understanding of the mechanisms behind pregnant hypertensive disorders.

Genetic factors substantially impact the development of PE and can affect the activity of liver enzymes by inducing endothelial dysfunction, inflammatory responses, oxidative stress, and immunological modulation [61]. Therefore, gaining a comprehensive understanding of these genetic associations is essential for enhancing the management and results of PE. It is also crucial to identify individuals at high risk of secondary complications of PE and further develop focused therapeutic approaches.

### 1.3. The Role of HIV Infection and Antiretroviral Therapy in the Development of Pre-Eclampsia

**Figure 1:**
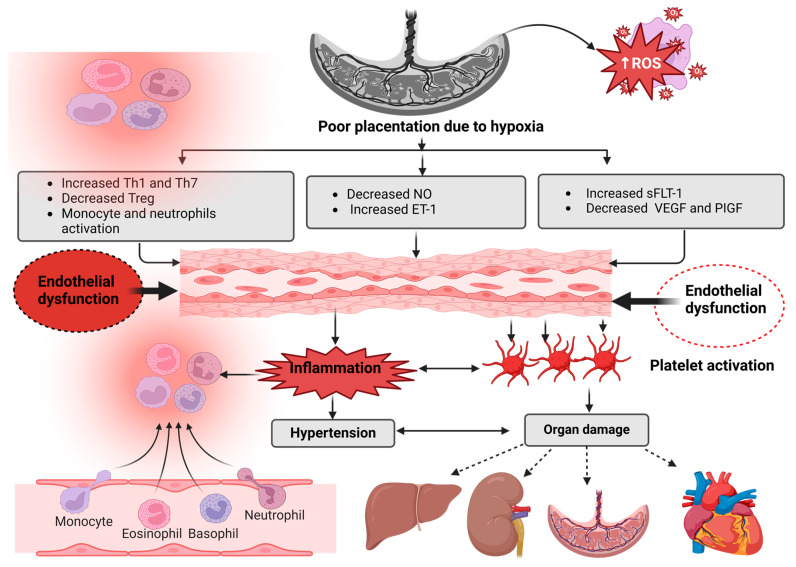
The contribution of immune and angiogenic system dysregulation plays a significant part in the pathophysiology of PE. ROS: reactive oxygen species, ET-1: endothelin-1, Th: T-helper cells, Treg: regulatory T-cell, sFLT-1: soluble fms-like tyrosine kinase, VEGF: vascular endothelial growth factor, PIGF: placental growth factor, and NO: nitric oxide. Adapted from [64]. Created in BioRender, https://BioRender.com/j02h242 (accessed on 5 November 2024).

The co-occurrence of HIV infection and PE is a rising maternal health concern driven by the shared occurrence of these conditions, especially in South Africa [62]. More recently, Modjadji et al. (2023) have shown that PWLWHIV on ART has an increased risk of cardiometabolic disorder [63]. Altogether, this, with PE, may compromise maternal and foetal health. Therefore, understanding the relationship between HIV and PE includes examining the immunological contribution, as shown in Figure 1. Poor placentation due to placental hypoxia, characterised by oxidative stress, impairs the angiogenic system, T-helper, and immune cells and causes vascular injury. These can be due to increased ET-1, a critical vasoconstrictor, sFLT-1, Th 1, and -7. Altogether, these contribute to endothelial dysfunction; in response, the body promotes platelet activation, further resulting in inflammation, organ damage, and hypertension or PE (Figure 1).

HIV infection alters immune systems and promotes a pro-inflammatory environment by producing TNF-α, IL-6, and IL-1β, thus resulting in inflammation and contributing to the pathophysiology of PE [65,66]. These cytokines are crucial in the inflammatory processes associated with HIV infection and PE. They worsen endothelial dysfunction and vascular abnormalities, which are important characteristics of PE [67]. The virus targets CD4+ T lymphocytes, resulting in their depletion and weakened immune function [68]. HIV plays a significant role in the pathogenesis of PE; ART-naïve individuals living with HIV (ILWHIV) have a reduced risk of developing PE due to the suppressed immune system’s necessary inflammatory response promoting PE development [62]. Several researchers have demonstrated the undesirable effect of ART and its contribution to the development of PE. Notably, these researchers reported that ART induces oxidative stress and endothelial dysfunction, both of which contribute to the pathogenesis of PE [69,70]. Therefore, it is important to understand the correlation between HIV, ART, and PE to improve maternal and newborn health outcomes in populations with a high prevalence of HIV. This information can improve the prevention of associated complications and management strategies for PWLWHIV.

Immunological systems contribute to the progression of pregnancy and HIV infection [71]. A cohort study conducted in South Africa found that pro-inflammatory cytokines like interferon gamma-induced protein 10 (IP-10) were more notable in PWLWHIV than in HIV-negative women [72]. Additionally, the same study reported significantly higher levels of Th1 cytokines, such as IL-12 and interleukin-12-protein 70 (IL-12p70), Th2 cytokine IL-5, and Th17 cytokine IL-17A in PWLWHIV than the HIV-negative group. The above study suggests that maternal HIV and ART use is associated with distinct systemic cytokine profiles throughout pregnancy, which can exacerbate the progression of PE. Therefore, it is more evident that HIV and PE are associated with increased inflammation [73]. Vyas et al. 2021 reported that PWLWHIV exhibited elevated levels of IL-6, TNFα, soluble CD14, and intestinal fatty acid-binding protein (I-FABP) [74]. Altogether, these results suggest that the presence of HIV in pregnancy promotes inflammation, monocyte activation, and gut barrier dysfunction, potentially contributing to the onset and exacerbation of PE. While HIV predominantly impacts the immune system, it can also invade liver cells, resulting in liver damage and apoptosis [75]. This is partly due to persistent immunological activation and inflammation [76]. Hepatocyte damage and apoptosis can be caused by elevated levels of pro-inflammatory cytokines, such as TNF-α and IL-6 [38,77]. Similar findings have also been reported in animal models of liver disorders [78]. This suggests that inhibiting inflammation can ameliorate liver damage and associated complications. Specific ART treatments, especially those containing PI, can result in hepatotoxicity, which manifests as increased levels of liver enzymes and liver damage [79]. ILWHIV are more susceptible to co-infections, such as hepatitis B and C, which can worsen liver damage and contribute to elevated liver enzyme levels [80]. Therefore, secondary complications among these populations must be curbed to prevent any manifestations of liver dysfunction.

### 1.4. The Role of HIV and ART in Liver Function

**Figure 2:**
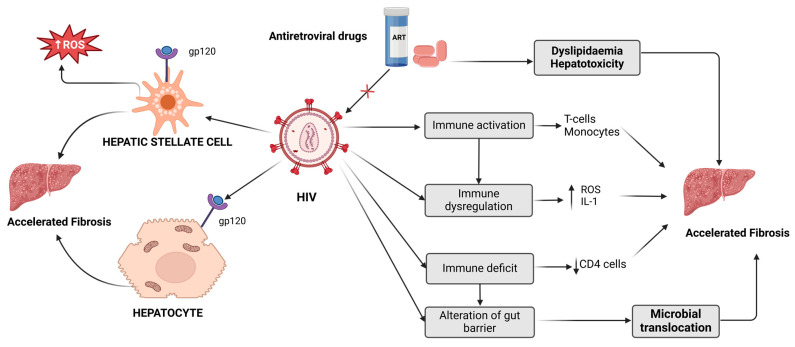
The impact of HIV and ART on the development of liver fibrosis. X: indicates inhibition, ART: antiretroviral therapy, ROS: reactive oxygen species, gp120: glycol-protein 120, CD4: cluster of differentiation-4, and IL-1: interleukin-1. Adapted from [98]. Created in Biorender, https://BioRender.com (accessed on 5 November 2024).

ART is an essential treatment for ILWHIV, since it suppresses the reproduction of the virus and enhances the functioning of the immune system [81]. ART achieves this by reducing inflammation [82]. This is important, because HIV infection is linked to persistent immunological activation and inflammation, which leads to various complications, such as CVD and liver damage [83]. However, ILWHIV on ART can also present with detrimental side effects, specifically on hepatic enzymes, which are markers for liver function [84]. Increased concentrations of these hepatic enzymes may suggest hepatic inflammation or injury, resulting in hepatitis or liver fibrosis [85]. ART categories, including nucleoside reverse transcriptase inhibitors (NRTIs), non-nucleoside reverse transcriptase inhibitors (NNRTIs), and protease inhibitors (PIs), are associated with elevated liver enzyme levels [86,87]. PIs and NNRTIs impair liver enzymes, including ALT and AST. ART can induce hepatotoxicity, which accelerates fibrosis (Figure 2); this condition is characterised by hepatic injury from drug exposure [88,89]. Additionally, as the ART improves, studies show that ILWHIV has elevated incidences of liver conditions such as viral hepatitis, alcohol-related liver disease, drug-induced liver damage, non-alcoholic fatty liver disease (NAFLD), and non-alcoholic steatohepatitis (NASH) [90,91,92]. A study by Chwiki et al. (2017) reported an increased level of ALT and AST in males living with HIV on ART [79]. Also, a study by Shiferaw et al. 2016 discovered a significant occurrence of liver enzyme abnormalities, mainly elevated ALT levels in patients on HAART compared to HAART-naïve [93]. The elevated ALT and AST levels were reportedly associated with viral hepatitis, opportunistic infections, CD4 count, and male gender. Drug-induced hepatotoxicity among ILWHIV and ART refers to the inherent toxic effects induced by ART on liver cells, mainly the hepatocytes [89,94]. These ARTs can potentially induce mitochondrial toxicity, altering the normal functioning of hepatocytes and resulting in the accumulation of fat in the liver [95]. For instance, stavudine and zidovudine that belong to NRTIs inhibit the function of DNA polymerase-γ, a crucial enzyme responsible for replicating and repairing mitochondrial DNA [96]. This inhibition leads to impaired functioning of the mitochondria, resulting in oxidative stress and damage to the liver cells [97].

ART contributes to increased liver enzymes through different pathways, including direct impact on the liver, immune system reconstitution, mitochondrial toxicity, and genetic factors [99]. For instance, immune reconstitution inflammatory syndrome (IRIS) occurs when ART improves the immune system, especially in HIV individuals with hepatitis, as the immune response may exacerbate hepatic inflammation and further elevate liver enzymes [100,101]. Additionally, ART in pregnant women with PE seems to have a discordant effect on liver function [102]. The information presented in Table 1 gives an overview of different studies that examine the effects of ART on liver function in PWLWHIV with and without PE. The presented research was conducted in various countries, including the United Kingdom, Ireland, Brazil, France, South Africa, and Nigeria. This provides a wide geographical reach, allowing for an understanding of how these complications may differ across different populations and healthcare systems. The sample size has also varied, with the smallest being 21 individuals to a maximum of 5748. Most studies have reported increased levels of liver enzymes, including AST, ALT, and ALP, in PWLWHIV, especially those actively receiving ART. Additionally, Joy et al. (2019) and Tamuno-Boma et al. (2023) revealed substantial increases in the ALT, AST, and ALP levels in PWLWHIV on ART compared to the ART-naïve group and HIV-negative group [103,104]. In contrast, Huntington et al. (2015) observed no statistically significant change in ALT levels among PWLWHIV on ART but confirmed the risk of LEE. Other researchers also showed no significant differences in liver function enzyme in PWLWHIV on ART compared to the counterpart group [105,106]. It is worth noting that participants did not have PE in all these studies. Yet, Maharaj et al. (2017) discovered no notable changes in ALT and AST levels between the PWLWHIV with PE and the HIV-negative group with PE [107]. However, the level of gamma-glutamyl transferase significantly increased in PWLWHIV with PE compared to the HIV-negative group. These suggest that the liver dysfunction in this group may be attributed to PE status rather than HIV and ART. On the other hand, Ouyang et al. 2009 reported a significant decrease in baseline liver enzyme elevation in PWLWHIV on nevirapine compared to NPWLWHIV [108]. According to Delicio et al. 2018, the use of NVP, nelfinavir, and atazanavir ART regimens was associated with an increased risk of liver function test abnormalities [109]. These results show that, while ART reduces the viral load, additional factors, including PE status, must be considered in managing liver function-associated complications. The evidence gathered in this study suggests that HIV and ART independently impair liver function (Table 1). Among women living with HIV without PE coexistence, the results highlight the impact of ART on liver enzyme levels. For instance, a study by Tamuno-Boma et al. (2023) revealed that women living with HIV on ART, whether pregnant or not, are more prone to liver dysfunction [103]; this warrants close monitoring and cautious care of this group of patients. There is a lack of evidence investigating the influence of PE on liver function calls for research to focus on this area to assess liver function in PWLWHIV with PE, especially in South Africa, where the prevalence of HIV is very high. This evidence is crucial, since PE can independently impair liver function, irrespective of HIV or ART administration [22]. The complex relationship between HIV, PE, liver function, and ART in pregnancy requires more investigation. The conflicting findings among previous studies emphasise the need for more focused research that longitudinally examines the impact of both PE and ART on liver function in HIV patients. A thorough understanding of these associations is crucial for enhancing ART regimens to minimise liver damage, especially in PWLWHIV with PE.

## 2. Limitations and Recommendations

Currently, there is limited evidence about the effect of ART on liver function among PWLWHIH with the co-existence of PE. This is evident in the lack of studies from databases such as PubMed and Scopus. This review did not highlight the exact class of ART regimens associated with liver dysfunction. Various forms (PI, NRTI, and NNRTIs) were used in individual studies, with others not specifying the regimen. Integrase strand transfer inhibitors (INSTIs) are currently used as first-line ART due to their superior efficacy, tolerability, minimal drug–drug interaction, and high genetic barrier to resistance [112]. The evidence about the effect of INSTIs such as dolutegravir has been observed among ILWHIV. For instance, the AST level was decreased while GST increased following the dolutegravir regimen in ILWHIV [113]. While dolutegravir reduced the HIV viral load and increased the CD4+ count compared to efavirenz, there was no significant difference in liver function test (AST and ALT) in both groups [114]. Altogether, these studies suggest some potential benefits of INSTI regimens in reducing liver dysfunction. However, there is limited evidence about its effect on liver function in pregnancy and PE. Geographic and population bias also contribute to the inconsistencies in the effect of ART regimens. The generalisability of the findings and conclusions may be limited to other populations, particularly those in regions with distinct healthcare systems, socio-economic conditions, and HIV and PE prevalence rates. Moreover, most of the studies analysed were cohorts, which limits the evaluation of the long-term impacts of HIV, ART, and PE on the health outcomes of both mothers and the transmission to children.

## 3. Conclusions and Recommendations

The evidence reviewed in this study showed discordant results about the effect of ART on liver function, especially in PWLWHIV compared to NPWLHIV. Other studies showed a significant increase in LEE or no difference, while others showed a decrease in LEE following ART. Similarly, among those with PE on ART, there were no differences in AST and ALT; however, gamma-glutamyl transferase increased compared to HIV-negative. Lastly, the risk of unexplained LEE was lower with PE on NNRTI than with PI-based regimens. Although HIV is often linked to elevated ALT, AST, and ALP, research on these liver enzymes in PWLWHIV in coexistence with PE remains limited, as evidenced by our finding that only two studies had PE. More importantly, this will enable proper care for PWLWHIV with PE. Therefore, future research should investigate the effect of ART on liver function in PWLWHIV and PE, especially in countries considered HIV endemic, like South Africa. Additionally, longitudinal studies are necessary to comprehend the implications in the long term among this population. Among the studies reviewed, none has explored the INSTI forms on liver function in PWLWHIV in co-existence with or without PE. Therefore, this calls for future studies to explore INSTI on pregnant women, focusing on liver function. Lastly, future studies should incorporate liver function tests in routine assessments during the maternal prevention of mother-to-child transmission program, thus reducing maternal and foetal adverse effects. Conducting future studies in South Africa can improve healthcare professionals’ capacity to give comprehensive care to PWLWHIV and PE.

## Figures and Tables

**Table 1:** Summary of studies on the effect of antiretroviral therapy in HIV-pregnant women with or without PE.

Authors, Year	Country	Study Design	Range/Mean Age (Years)	Sample and Population	Summary of Findings
Hungtigton et al. 2015 [110]	United Kingdom and Ireland	Cohort	29–39	3815 PWLWHIV on ART.	There was no significant difference in ALT in pregnant women on ART.
Joy et al. 2019 [104]	Nigeria	Cohort	20–40	30 PWLWHIV on HAART 30 HAART-naïve PWLWHIV 30 HIV-negative.	Significant increase in ALT, AST, and ALP in PWLWHIV on HAART compared to the ART-naïve or negative at the 1st, 2nd, and 3rd trimesters.
Maharaj et al. 2017 [107]	South Africa	Cohort	24.8 ± 5.3 28.7 ± 7.3 24.6 ± 6.4 28 ± 6.4	53 HIV-negative with PE 45 PWLWHIV with PE 50 normotensive and HIV-negative 45 normotensive and PWLWHIV.	There is no significant difference in AST and ALT between PE-PWLWHIV and HIV-negative. The gamma-glutamyl transferase increased in PWLWHIV with PE compared to negative.
Onyeka et al. 2016 [105]	Nigeria	In vivo experimental	30 ± 3.0	21 PWLWHIV on ART 25 NPWLWHIV.	There were no significant differences between the pregnant and non-pregnant groups in the AST, ALP, and ALT levels.
Sibiude et al. 2019 [111]	France	Cohort	<25 25–39 ≥40	5748 PWLWHIV on ART, of which 147 had PE.	Among PWLWHIV on ART at conception, the risk of unexplained LEE was lower with NNRTI compared to PI-based regimens.
Tamuno-Bona et al. 2023 [103]	Nigeria	Cross-sectional	15–60	83 PWLWHIV on ART 82 NPWLHIV on ART 84 PWLWHIV-negative 81 NPHIV-negative.	Significantly higher ALT, AST and ALP levels in PWLWHIV compared to non-PWLWHIV. Lower AST, ALT, and ALP in HIV-negative pregnant women compared to non-pregnant HIV-negative.
Ouyang et al. 2010 [106]	United States	Prospective cohort	27.84 28.02	218 PWLWHIV on nevirapine (NVP) 1011 non-NVP PWLWHIV.	No significant liver elevation was observed in the NVP compared to the non-NVP group.
Ouyang et al. 2009 [108]	United States	Prospective cohort	27.99 35.96	1229 PWLWHIV on NVP 821 NPWLWHIV on NVP.	Significant decrease in baseline in liver enzyme elevation in PWLWHIV compared to NPWLWHIV.
Delicio et al.2018 [109]	Brazil	Cohort	13–46	801 PWLWHIV with 793 on known ART and eight on unknown ART.	NVP, nelfinavir and atazanavir regimens increased the risk of liver abnormalities.

PWLWHIV: pregnant women living with human immune virus; NPWLWHIV: non-pregnant women living with human immune virus; NVP: nevirapine; HAART: highly active antiretroviral therapy; ART: antiretroviral therapy; AST: aspartate aminotransferase; ALT: alanine aminotransferase; ALP: alkaline phosphatase.

## Data Availability

Not applicable.
